# Nanoparticles influence droplet formation in a T-shaped microfluidic

**DOI:** 10.1007/s11051-013-2128-x

**Published:** 2013-11-30

**Authors:** Ruijin Wang

**Affiliations:** Zhejiang University of Science and Technology, Hangzhou, 310023 China

**Keywords:** Droplet formation, Nanoparticles, T-shaped microfluidic, Numerical simulation, Fluid dynamics

## Abstract

Droplet formation in the presence of nanoparticles was studied in a T-shaped microfluidic device numerically. Nanoparticles in continuous phase did not influence droplet formation dynamics obviously. Contrarily, the presence of nanoparticles in dispersed phase will influence evidently droplet formation dynamics, likely reasons are the accumulation of nanoparticles at the liquid–liquid interface leading to the variation of interfacial tension and the anisotropy of nanoparticles’ movement at interface. The droplet size decreases almost linearly with increasing of the volume fraction of nanoparticles in dispersed phase when the volume fraction of nanoparticles not exceeding a critical value (about 0.2 %), because very high concentration of nanoparticles results in particle aggregation so as to not decrease interfacial tension so obviously any more. A complicated mechanism of temperature influences on droplet formation may exist combining the variations of effective viscosity and interfacial tension. Discussions on microscopic mechanism of droplet formation in the presence of nanoparticles were carried out.

## Introduction

Droplet-based microfluidics, as one of the main flow forms of microfluidics, have greatly interested many researchers, and have been widely used in the areas of nanomaterials preparation, pharmaceutical analysis, protein engineering, and so on. Research on droplet dynamics behavior mainly focus on mechanism of droplet formation, control of droplet size (John et al. 2008; Wang et al. 2003; Branzes et al. 2009). The T-shaped junction microchannels have been used for droplets formation, fission, fusion, emulsions preparation, and microscale mass transportation enhancement (Li et al. 2012; Baround et al. 2010). In T-junction microchannels, the dispersed phase liquid is discretized into separate elements by immiscible continuous phase liquid. This process involves complex mechanism, which derives from the force competition among the interfacial tension, viscous shearing, pressure drop and possible force perturbations out of the system. Menech et al. (2008) described the results of a numerical investigation on the dynamics of breakup of streams of immiscible fluids in a microfluidic T-junction. Three distinct regimes of formation of droplets can be seen: squeezing, dripping, and jetting. In squeezing regime, the breakup process is driven chiefly by the buildup of pressure upstream of an emerging droplet, the breakup dynamics and the droplet size are influenced only weakly by the capillary number. The dripping regime is also significantly influenced by the constrained geometry; these effects modify the scaling law for the size of the droplets derived from the balance of interfacial and viscous stresses. Finally, the jetting regime sets in only at very high flow rates, or with low interfacial tension, i.e., higher values of the capillary number, similar to the unbounded case. Various approaches of control of droplet size are described in Refs. (Sang et al. 2009; Gupta and Kumar 2010; Zhang et al. 2009; Gong et al. 2010; Muradoglu and Tryggvason 2008) by adjusting viscosity ratio, flow rate ratio, T-junction geometries, capillary number, wettability of the channel, temperature, surfactants, and external force field.

It was known to us that nanoparticles will influence the behaviors in microfluidic system (Neculae et al. 2012). And some research fruits in droplet hydrodynamics were achieved in the past decade. Priest et al. 2011 have studied the influence of nanoparticles on the early stages of Pickering emulsification using a microfluidic chip. Droplet formation in microfluidic channels is almost insensitive to the presence of nanoparticles in the continuous phase. A fluid named nanofluid, in which 15 nm TiO_2_ nanoparticles are dispersed evenly, was found to exhibit considerably smaller surface tension and oil-based interfacial tension than those of the base fluid itself (Murshed et al. 2008a). Experimental results also demonstrated that surface and interfacial tensions of the nanofluid decrease with increasing temperature. The dispersion of a small volume fraction of nanoparticles significantly increases the droplet size of deionized water formed at a microfluidics T-junction and the droplet size was found to increase nonlinearly with increasing temperature. Fischer (Fischer et al. 2010) investigated the thermal transport of liquid–liquid flow through microchannels for different flow conditions, and a positive effect of Al_2_O_3_ nanoparticles on the heat transfer performance in microchannels was detected. This is mainly caused by the fact that nanoparticles lower the effective interfacial tension of the fluids. Lower surface tension results in larger droplet size. Sheu et al. 2010 investigated the two-phase flow formation process of water-based Fe_3_O_4_ ferrofluid (dispersed phase) in a silicon oil (continuous phase) flow under various operational conditions, four main two-phase flow patterns as droplet flow, slug flow, ring flow, and churn flow are observed in the experiments. The droplet shape, size, and formation mechanism were also investigated under different capillary number. The flow pattern transiting from droplet flow to slug flow appears under an operational condition (flow rate ratio being less than one and dimensionless droplet size being less than one). The power law index that related dimensionless droplet size to flow rate ratio was 0.36. The effect of an applied magnetic field on the droplet size and the velocity field was investigated experimentally and numerically (Liu et al. 2011). The size of droplet increases with increasing magnetic field strength. The sensitivity of the droplet size on the magnetic field depends on the flow rates of both continuous and the dispersed fluids. The higher the magnetic bond number, the larger is the volume of the formed droplet. In the absence of the magnetic field, a couple of opposite flow appears as results of the interaction between the pressure drop, viscous drag force, and interfacial tension. In the presence of a magnetic field, the ferrofluid tip is pulled forward due to the additional magnetic force. The thread and the tip become longer resulting in a longer formation time. The results show that using an external homogenous magnetic field is an alternative to control the droplet size of a ferrofluid emulsion.

It however remains unclear, how the nanoparticles in fluid influence the droplet formation mechanism and droplet size so far. We intend to investigate the hydrodynamics behavior of droplets containing nanoparticles in this paper numerically. Firstly, nanoparticles’ location and velocity are calculated after considering the interaction between particle–particle and particle–fluid, and then the interfacial tension, interfacial curvature, density distribution, and viscosity distribution can be obtained. Fluid containing nanoparticles is regarded as a single phase, we can solve the whole flow field using traditional VOF method. Secondly, processes of droplet formation would be numerically simulated in the presence of nanoparticles in continuous phase or dispersed phase, in order to investigate the influence of particle concentration, particle diameter, and temperature. Finally, the essential reasons of influence on interfacial tension, as well as the mechanism of interfacial tangent slip, are discussed.

### Geometric model and parameter settings

**Fig. 1:**
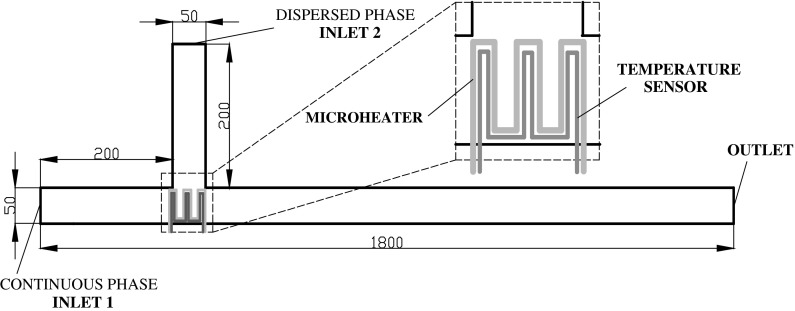
Schematic diagram of geometric models for simulation (Unit: micrometer)

**Fig. 2:**
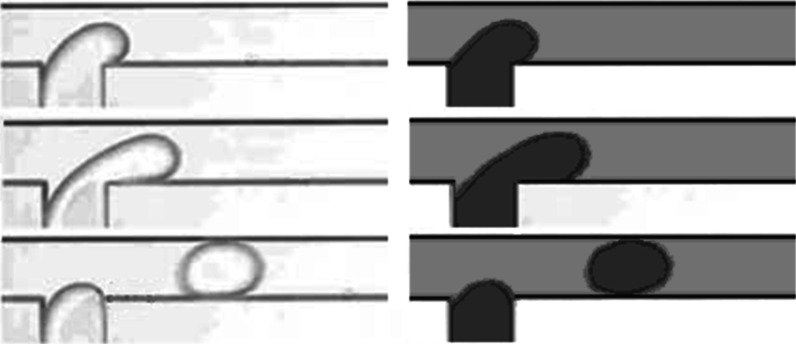
Droplet formation process (*right*: our simulation results, *left*: experimental results of Van Der Graaf et al. (2006), Reprinted with permission from Van Der Graaf et al. (2006). Copyright 2006 American Chemical Society

**Fig. 3:**
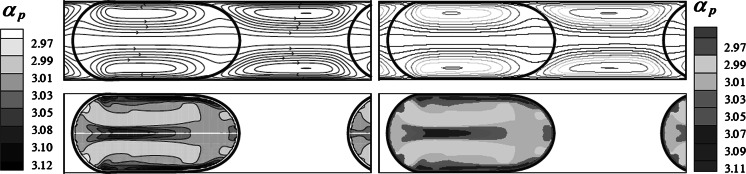
Streamlines in droplet laden flow (*Top*) and nanoparticles distribution in droplet (*Bottom*). *Right*: our numerical results, *Left*: numerical results of Fischer et al. (2010). Reprinted with permission from Fischer et al. (2010). Copyright © 2010 by ASME Here, $\alpha_{\text{p}}$ is particle volume fraction

The microchannel under investigation is presented in Fig. 1. The inlets 1 and 2 are injected with deionized water as continuous phase and silicone oil as dispersed phase, respectively. The outlet for drainage is long enough off the junction, so we are able to simulate long droplet strain at high capillary number (ratio of viscosity force and interfacial force). Uniform inlet velocities are set at the two inlets, while the outflow is set at the outlet. The reference atmospheric pressure is given at the inlet 1, and the no-slip condition is used at the walls, with a hydrophobic contact angle of 105°, which is identical to those in an untreated dimethylsiloxane, i.e., PDMS. The inlet for dispersed fluid and the main channel for continuous fluid are pre-filled with continuous phase. At the T-junction microheater and temperature sensors are hypothetically embedded to control and measure the local temperature (Figs. 2, 3).

The width and depth of the channel are 50 and 30 μm. In view of full-developed velocity profile obtained before the continuous phase and dispersed phase meeting at the junction, 200 μm is necessary from inlet to T-junction. The drainage is set to be 1,550 μm off the junction, so that it is long enough to simulate long droplet strain.

The settings of base fluids properties: the viscosity, density, specific heat, and thermal conductivity of continuous phase are 1.003 g/m s, 0.9982 g/cm^3^, 4,180 J/kg K, 0.6 W/m K respectively; the viscosity, density, specific heat, and thermal conductivity of dispersed phase are 50 g/m s, 0.950 g/cm^3^, 1,630 J/kg K, 0.16 W/m K respectively. Density and specific heat of Fe_3_O_4_ nanoparticle are 5.18 g/cm^3^ and 937 J/kg K. The interfacial tension between deionized water and silicone oil is 30 mN/m at 25 °C.

### Numerical model

The numerical model includes the continuity equation, Navier–Stokes equation which is still valid in current microchannel flow, and continuity equation for volume fraction.

1 $$\nabla \cdot V = 0$$

2 $$\rho \left[ {\frac{\partial V}{\partial t} + \left( {V \cdot \nabla } \right)V} \right] = - \nabla p + \mu \nabla^{2} V + F$$

3 $$\frac{{\partial \alpha_{{}} }}{\partial t} + V \cdot \nabla \alpha_{{}} = 0$$

where $p$ is the pressure, $\mu$ is the dynamic viscosity, $\rho_{{}}$ is the density, $\alpha_{{}}$ is volume fraction of continuous phase or dispersed phase, $F$ is the source term to account for the interfacial tension force. In this source term, the VOF model is tightly connected, i.e., in a computational cell, the density and the viscosity of the two-phase mixture read as

4 $$\rho = \alpha_{1} \rho_{1} + \alpha_{2} \rho_{2}$$

5 $$\mu = \alpha_{1} \mu_{1} + \alpha_{2} \mu_{2}$$

where $\alpha_{1}$, $\alpha_{2}$ are the volume fractions of continuous phase and dispersed phase respectively, and must satisfy $\alpha_{1} + \alpha_{2} = 1$. $\rho_{1}$, $\rho_{2}$ are the density of continuous phase and dispersed phase.

However, we must consider a condition for the Eq. (2) with no-slip boundary being valid, e.g., the Knudsen number $Kn = {\ell \mathord{\left/ {\vphantom {\ell L}} \right. \kern-0pt} L}$ is less than 10^−3^, where $\ell$ is the mean free path of molecules. Here $\ell$ is about 0.001–0.08 μm, the length scale $L$ is 50 μm, then the Knudsen number is about 1–80 × 10^−5^.

The source term correlated with interfacial tension based on the continuum surface force (CSF) model can be calculated as (Fischer et al. 2010):

6 $$F = \int\limits_{{S^{'} }} {\left( {\sigma \kappa \bar{n} + \nabla_{s} \sigma } \right)} \delta \left( {x - x^{'} } \right)dS$$

where $\sigma$ and $\kappa$ denote interfacial tension and the curvature of the freely deformable interface. $\bar{n}$ is the normal vector to the interface, $\nabla_{s}$ is the gradient operator tangential to the interface, and the Dirac distribution $\delta \left( {x - x^{'} } \right)$ localizes the effects only at the interface, and $S^{\prime}$ is the interface area. $\kappa$ can be computed by the contact angle.

The nanoparticle continuity and energy equations are derived from Buongiorno (2006). In our simulation, the nanofluid (uniform suspension of nanoparticles in based fluid) is treated as a single phase, and is considered to be a dilute mixture. Brownian diffusion and thermophoresis, which is the particle motion under the influence of a thermal gradient, can be regarded as the only slip mechanisms for nanoparticle transport at low Reynolds number in microflow. They are incorporated into the nanoparticle transport equation as follows:

7 $$\frac{{\partial \alpha_{\text{p}} }}{\partial t} + V \cdot \nabla \alpha_{\text{p}} = \nabla \cdot \left[ {D_{B} \nabla \alpha_{\text{p}} + D_{T} \frac{\nabla T}{T}} \right]$$

where $\alpha_{\text{p}}$ denotes the particle volume fraction. Brownian diffusion and thermophoresis are described by the first and second terms on the right hand side. $D_{B}$ and $D_{T}$ are Brownian diffusion coefficient and temperature-dependent thermal diffusion coefficient, respectively. In the nanofluid, the coefficients read as:

8 $$D_{\text{p}} = \frac{{k_{B} T}}{{3\pi \mu_{b} d_{\text{p}} }},\quad D_{T} = \beta \frac{{\mu_{\text{n}} }}{{\rho_{\text{n}} }}\alpha_{\text{p}}$$

where $k_{B}$ denotes Boltzmann’s constant, $\mu_{\text{b}}$, $\mu_{\text{n}}$viscosity of based fluid and nanofluid respectively, $\rho_{\text{n}}$ is density of nanofluid, $d_{\text{p}}$ is particle diameter, and $\beta$ is a proportionality factor. In the bulk fluid (based fluid), both are set to be zero.

In the presence of nanoparticles, the energy equation takes below form:

9 $$\rho_{\text{n}} c_{\text{n}} \left[ {\frac{\partial T}{\partial t} + V \cdot \nabla T} \right] = \nabla \cdot k\nabla T + \rho_{\text{p}} c_{\text{p}} \left[ {D_{B} \nabla \alpha_{\text{p}} \cdot \nabla T + D_{T} \frac{\nabla T \cdot \nabla T}{T}} \right]$$

Here, $\rho_{\text{p}} ,c_{\text{p}}$ are the nanoparticle density and specific heat. The entire term in brackets on the right hand side accounts for thermal transport due to particle motion. An order of magnitude analysis reveals that this term is negligible, and the energy equation becomes identical to that of a pure fluid (Fischer et al. 2010). It follows that the nanoparticles affect the heat transfer in the nanofluid only by their influence on the thermophysical properties. The property field varies not only between the two liquids, but also within the nanofluid as it is a function of particle concentration. The density and specific heat of the nanofluid can be calculated using standard mixture laws:

10 $$\rho_{\text{n}} = \alpha_{\text{b}} \rho_{\text{b}} + \alpha_{\text{p}} \rho_{\text{p}}$$

11 $$c_{\text{n}} = \frac{{\alpha_{\text{b}} c_{\text{b}} \rho_{\text{b}} + \alpha_{\text{p}} c_{\text{p}} \rho_{\text{p}} }}{{\rho_{\text{n}} }}$$

$\rho_{\text{b}} ,\rho_{\text{p}}$ denote density of based fluid and particles, $c_{\text{b}} ,c_{\text{p}}$ are specific heat of based fluid and particles, respectively. There is still a lack of accurate theoretical models for the prediction of the viscosity and thermal conductivity of nanofluids. Normally, empirical laws (Murshed et al. 2008a) to predict the viscosity and thermal conductivity of nanofluids read as:

12 $$\mu_{\text{n}} = \mu_{\text{b}} \left( {1 + 18.8\,\alpha_{\text{p}} } \right)$$

13 $$k_{\text{n}} = k_{\text{b}} \left( {1 + 4.97\,\alpha_{\text{p}} } \right)$$

$k_{\text{n}}$, $k_{\text{b}}$ denote the thermal conductivity of nanofluid and thermal conductivity of the based fluid, respectively.

Similarly, there is no interfacial tension model for the nanofluid. In our simulation, the interfacial tension was calculated with following equation:

14 $$\sigma_{0} = \sigma_{ 1} + \sigma_{2} - 1.35\sqrt {\sigma_{1} \sigma_{2} }$$

where $\sigma_{1}$, $\sigma_{2}$ are the surface tension of continuous phase and dispersed phase with nanoparticles, respectively. After simplifying and over-fitting of the data in relevant Ref. (Murshed et al. 2008a, b) it can be estimated by:

15 $$\sigma_{1,2} = \sigma_{01,02} \left( {1 - 7.38\;\alpha_{\text{p}} } \right)$$

In our simulation $\sigma_{01} ,\sigma_{02}$ were set to be 72.0 and 20.8 mN/m, and $\alpha_{\text{p}}$ is volume fraction at interface here.

It is known to all that interfacial tension and viscosity are temperature dependent, and interfacial tension can be calculated assuming a linear dependence on temperature, viscosity can be calculated assuming an index dependence on temperature.

16 $$\sigma - \sigma_{0} = - 0.06\Updelta T$$

17 $$\ln (\mu ) - \ln (\mu_{0} ) = - 0.017\Updelta T$$

### Validation with existing results

In order to verify the availability of the numerical model, we simulated numerically droplet formation process in a T-junction microchannel which was the same as that performed by Van Der Graaf and Nisisako (2006). After comparing with the experimental snapshot, we can confirm our numerical is suitable to simulate droplet formation process.

In the same way, nanofluid droplets laden microflow is simulated numerically to validate the numerical model in the presence of the nanoparticle. Comparing with the numerical results presented in Ref (Murshed et al. 2008b), we confirm the availability of our numerical model for the droplet laden microflow.

## Results and discussion

For the production of droplets, the process seems similar for squeezing, dripping, and stable jetting regime. The tip of the dispersed phase fluid intrudes into the main channel, and then the tip grows under the balance of interfacial tension, shearing force, and the pressure drop occurred between the tip and rear of the emerging droplet. When the emerging droplet marches downstream for a certain distance, the convex arc interface shrinks to a concave neck, finally ruptures and the droplet is released.

There are many important parameters of the continuous phase and dispersed phase that will affect droplet formation. Accordingly, the problem can be described in terms of following dimensional and physical parameters: interfacial tension $\sigma$, channel depth $h$, channel width $w_{c} ,w_{d}$, flow velocity $v_{c} ,v_{d}$, viscosity $\mu_{c} ,\mu_{d}$, density $\rho_{c} ,\rho_{d}$ (subscript *c* and *d* denote that of continuous phase and dispersed phase). Hence, we can define six independent dimensionless parameters based on the above mentioned parameters, $Q = \frac{{v_{d} w_{d} }}{{v_{c} w_{c} }}$, $Ca = \frac{{\mu_{c} v_{c} }}{\sigma }$, $\text{Re} = \frac{{\rho w_{c} v_{c} }}{{\mu_{c} }}$, $\lambda = \frac{{\mu_{d} }}{{\mu_{c} }}$, $\varLambda = \frac{{w_{d} }}{{w_{c} }}$ and $\varGamma = \frac{h}{{w_{c} }}$.

In this section the formation of the droplets, especially, the droplet size is investigated. The droplet diameter *D*_*d*_ is defined as droplet volume divided by channel width of continuous phase, channel width of dispersed phase and depth, i.e., $D_{d} = \frac{{V_{d} }}{{w_{c} w_{d} h}}$, here, $V_{d}$ is droplet volume, which can be exported after calculation, because most of the droplets in published experiments are longer than the width of continuous phase channel.

The inlet velocity of continuous phase is set to be 0.12–1.2 mm/s, and the flow rate ratio ( $Q$) ranges from 0.005 to 5. The capillary number ( $Ca$) is 0.004–0.04, the Reynolds number is about 0.006–0.06, the ratio of depth to width ( $\varGamma$) is 0.6, the channel width ratio for continuous phase to dispersed phase ( $\varLambda$) is 1. The viscosity ratio ( $\lambda$) is about 50 irrespective of nanoparticles suspending in based fluid. The particle volume fraction of the continuous phase is 0.2 %, and for the dispersed phase is 0.1–2 %.

The problem is solved using one set of continuity equations for the entire domain both inside and outside the droplets. Both continuous phase and dispersed are treated as incompressible with no external body forces as the influence of gravity can be regarded as negligible. This is justified by the fact that the Bond number is well below unity ( $Bo = \frac{{\rho_{c} gw_{c}^{2} }}{\sigma }$«1).

### Nanoparticle-dependent droplet size

Numerical simulations have been performed on the influence of droplet size of nanoparticle volume fraction with various flow rate ratio and capillary number. Theoretically, the viscosity influences interfacial tension and flow characteristics, and the viscosity of a nanofluid varies with increasing nanoparticle volume fraction. It is a pity, no theoretical model is available for the prediction of the effective viscosity of nanofluids as a function of particle volume fraction so far.

**Fig. 4:**
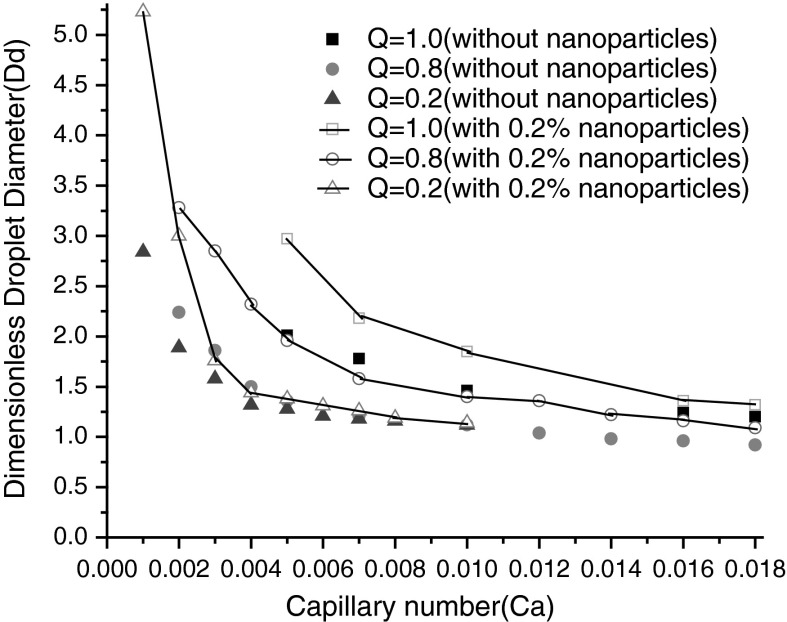
The effect of presence of 0.2 % nanoparticle in dispersed phase, that of Capillary number (Ca) and flow rate ratio(Q) on the dimensionless droplet diameter at a fixed *λ* = 50, *Λ* = 1, *Γ* = 0.6

**Fig. 5:**
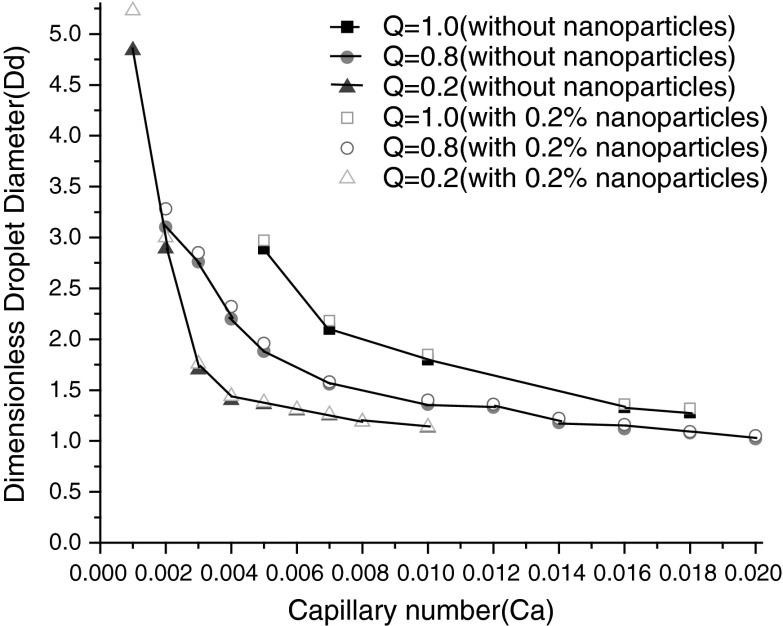
The effect of 0.2 % nanoparticle in the presence of continuous phase, that of Capillary number (Ca) and flow rate ratio (Q) on the dimensionless droplet diameter at a fixed *λ* = 50, *Λ* = 1, *Γ* = 0.6

There are many established results about the effects on the droplet size of Capillary number and flowrate ratio in the absence of nanoparticle in fluid (Li et al. 2012; Menech et al. 2008; Sang et al. 2009; Gupta and Kumar 2010; Sivasamy et al. 2011). To insight effect of the presence of nanoparticles in dispersed phase, numerical simulations are carried out at a fixed *λ* = 50, *Λ* = 1, *Γ* = 0.6, flow rate ratio *Q* is 1.0, 0.8, 0.2, respectively. Capillary number ranges from 0.001 to 0.02, for such small values of capillary numbers the droplet formation is in the squeezing (pressure dominated) regime, suggesting that interfacial tension dominates viscous stress. We can see from Fig. 4, nanoparticles in dispersed phase affect the droplet size obviously, especially at low Ca and Q, nearly 50 % reduction can be achieved. The reasons should be that, nanoparticles increase slightly the effective viscosity on the one hand, decrease violently the interfacial tension on the other hand. In the microscopic view, Brownian motion and thermophoresis cause nanoparticle enrichment at interface. In addition, Brownian motion is anisotropic, thermal motion of nanoparticle takes place more effortless tangential to interface than that of normal to interface because of the constraint of interfacial tension. However, in the presence of nanoparticles in continuous phase, less than 10 % reduction of the droplet size come into being even at very low Ca and *Q* (see Fig. 5), because the variation of viscosity ratio (*λ*) is not so great, that only a slight influence has been exerted on interfacial tension and droplet formation process.

### Influence of particle volume fraction and particle diameter

**Fig. 6:**
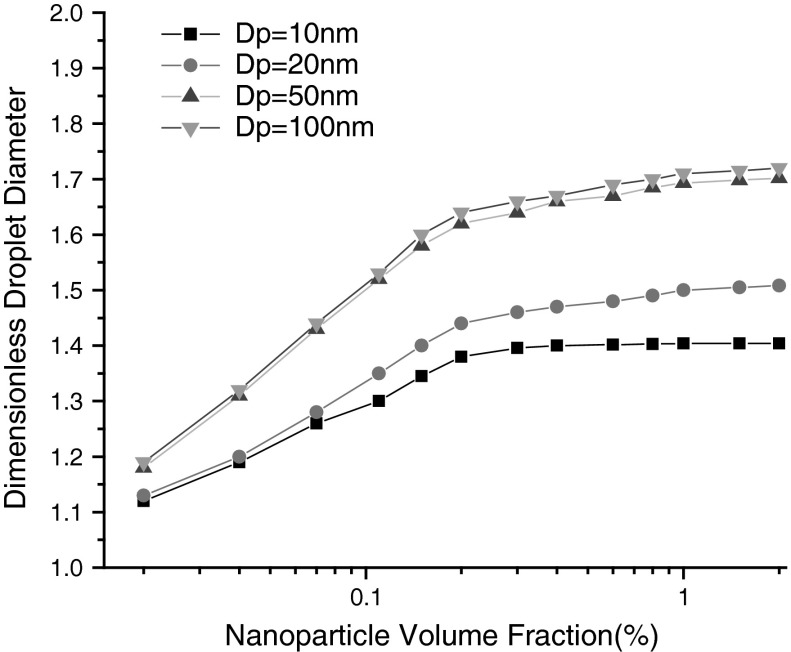
The effect of volume fraction (*α*_p_) and particle diameter (*D*_p_) in the presence of dispersed phase on the dimensionless droplet diameter at a fixed *Q* = 0.8, Ca = 0.02, *λ* = 50, *Λ* = 1, *Γ* = 0.6

In order to study the influence of particle volume fraction (*α*_p_) on the droplet formation, numerical simulations were carried out when *α*_p_ ranges from 0.01 to 2 % at a fixed *Q* = 0.8, Ca = 0.02, *λ* = 50, *Λ* = 1, *Γ* = 0.6. We can see from Fig. 6, dimensionless droplet diameters increase greatly with the increase of *α*_p_ in dispersed phase when *α*_p_ being less than 0.2 %, while a trifle increase of droplet diameter with the *α*_p_ exceeds 0.2 %. In consideration of nanoparticles being as a dilute phase in dispersed fluid when *α*_p_ being less than 0.2 %, the interactions and aggregation of nanoparticles can be neglected. More nanoparticles contain in fluid, more interfacial tension reduction emerges, and greater droplets generate. On the contrary, the interactions and aggregation of nanoparticles can’t be neglected when *α*_p_ being greater than 0.2 %, interfacial tension reduction appears very small.

Calculations were performed in allusion to four particle diameters ranging from 10 to 100 nm. Figure 6 indicated that fine particle diameter results in relative small droplet, when particle is not coarser than 50 nm, and no obvious difference was observed between particle 50 and 100 nm. The accounts should be taken as: greater Brownian motion existence possesses when particle being tiny, greater reduction of interfacial tension could be produced, however, no more reduction of interfacial tension generates when the particle bigger than a critical value. Another interpretation is that the particles at interface can move further along interface curve than that of normal to interface, and the tangential slip could be aroused. All of above mentioned are the reasons of droplet diameter reduction.

### Temperature dependent droplet size of nanofluid

**Fig. 7:**
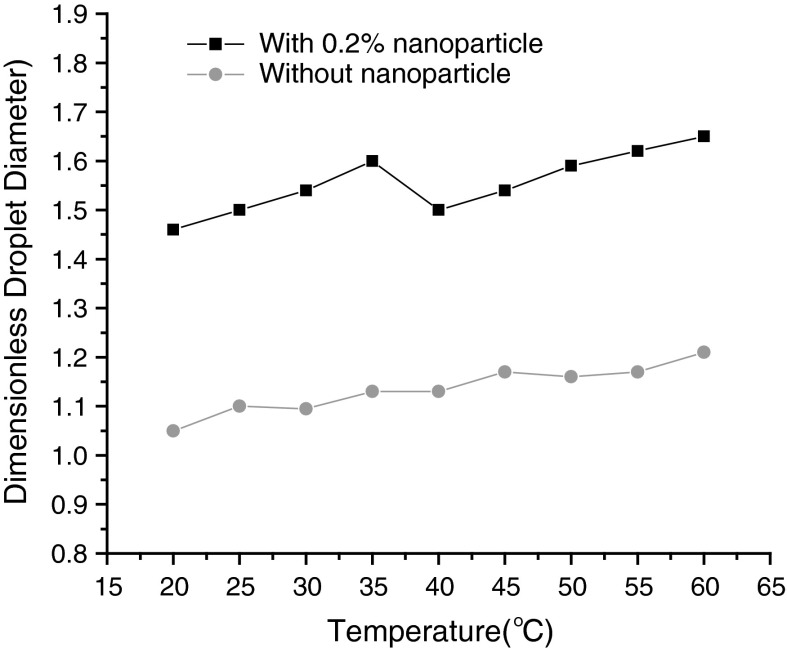
The effect of temperature (T) and nanoparticle in the presence of dispersed phase on the dimensionless droplet diameter at a fixed *Q* = 0.8, Ca = 0.02, *λ* = 50, *Λ* = 1, *Γ* = 0.6

It is worthwhile studying temperature-dependent droplet size of nanofluids for their potential droplet size control applications in microfluidic systems. In our work, the temperature dependence of droplet formation and size manipulation is numerically investigated at the microfluidic T-junction. Droplet formation of both silicone oil and silicone-based-nanofluid is characterized at different temperatures ranging from 20 to 60 °C. Figure 7 shows us the temperature dependence of the droplet size in a T-junction microfluidic device. It can be seen that the droplet sizes of nanofluids are larger than its base fluid and the dependence on temperature is more significant compared with the base fluid. Since the effective viscosity of the nanofluid does not change significantly with temperature, the formation of droplet is governed by the interfacial tension, which is much smaller for nanofluid than that of silicone oil.

A remarkable ravine can be seen at the curve for nanofluid in the temperature 35–45 °C. The mechanism is very complicated, because the integrated influence of particle movement and interaction should be taken into account to estimate effective viscosity and interfacial tension. At relative lower temperature the reduction of interfacial tension is more significant than that of viscosity (being drawn from Eqs. 12, 13), dimensionless droplet diameter increases with increase of temperature because of the decrease of the dominated interfacial tension. Nevertheless, comparison with interfacial tension the alteration of effective viscosity is relatively significant at higher temperature, the reduction of effective viscosity will be dominated and more apparent than that of interfacial tension.

A discussion on particle movement and interaction should be had with a microscopic perspective. Particle movement includes mainly Brownian motion and thermophoresis, and particle interaction includes particle–liquid interaction and particle–particle interaction. Brownian motion is efficient in redistributing the particles to the lowest total free energy at the interface. Moreover, an elevated temperature intensifies the Brownian motion and it is known that, the lower the cohesive energy, the smaller the interfacial tension. Furthermore, nanoparticles can be adsorbed at the liquid–liquid interface and function in similar way to the surfactants to reduce the surface tension or interfacial tension (Holt et al. 2010). Similar to the alteration of thermophysical properties (Muradoglu and Tryggvason 2008; Murshed et al. 2008b), the addition of a small amount of nanoparticles into the liquid can change obviously its surface properties such as interfacial tensions.

## Conclusion

The droplet formation process of silicone oil containing nanoparticles in deionized water as continuous phase was investigated for different flow conditions, such as volume fraction of nanoparticles, temperature, capillary number, and flow rate ratio. A comprehensive model was presented with a front tracking algorithm to capture multiphase microflow and interfacial effects by combining nanoparticle transport. With thorough parametric studies, we elaborated on the strong effect of interfacial tension on the droplet size in a microfluidic T-junction. We observed that the influence on interfacial tension of nanoparticles uniform suspension in silicone oil as dispersed phase strongly affects the droplet formation in low capillary number and Reynolds number microflow, likely reason is that the accumulation of nanoparticles at the liquid–liquid interface would cause the variation of interfacial tension and the anisotropy of particles’ movement at interface. Contrarily, nanoparticles in continuous phase did not influence droplet formation dynamics obviously. The droplet size decreases almost linearly with increasing of the volume fraction of nanoparticles in dispersed phase when the volume fraction of nanoparticles not exceeding a critical value (about 0.2 %), because very high concentration of nanoparticles decreases the interfacial tension not so obviously any more. The droplet size was found to increase nonlinearly with increasing temperature. The mechanism is very complicated, because the integrated influence of particle movement and interaction should be taken into account to estimate effective viscosity and interfacial tension. The Brownian motion and adsorption of nanoparticles at the interfaces were identified as mechanisms for reducing the interfacial tensions of nanofluid. It is also imperative to conduct more studies in order to elucidate the mechanisms for temperature-dependent interfacial properties, viscosity and droplet formation of fluids in the presence of nanoparticles.
